# Do Not Drink Poppers: A Case Report of Near Fatal Methemoglobinemia After Ingestion of Alkyl Nitrite

**DOI:** 10.7759/cureus.77190

**Published:** 2025-01-09

**Authors:** Marissa Valenzuela, Tammy Phan, Emmelyn Samones, W. Seth Dukes

**Affiliations:** 1 Emergency Medicine, Loma Linda University Medical Center, Loma Linda, USA

**Keywords:** amyl nitrite, methemoglobinemia, methylene blue, nitrite-containing poppers, poppers

## Abstract

A 23-year-old female presented to the Emergency Department (ED) with altered mental status and acute respiratory failure with hypoxia after ingesting poppers (alkyl nitrite) at a music festival. She received initial treatment at the event medical tent but deteriorated during transport. Upon arrival at the ED, she required intubation and received methylene blue treatment for suspected methemoglobinemia. Labs confirmed methemoglobinemia, and her blood appeared chocolate brown. Despite requiring vasopressors, her condition improved after methylene blue administration. Following treatment, her oxygen saturation and blood gas improved. She was discharged within 24 hours. Poppers are often recreationally inhaled at social events. We present a case where drinking poppers led to a near-fatal methemoglobinemia. The presentation was recognized and treated with methylene blue. It is important for the healthcare team to consider alkyl nitrites when a patient presents with cyanosis and hypoxia not improving above 85%. Administration of methylene blue can reverse methemoglobinemia and prevent fatalities.

## Introduction

Poppers, also known as alkyl nitrites, including amyl nitrite and butyl nitrite, are recreational drugs used at music festivals and in the nightlife scene for feelings of euphoria and relaxation [1]. Alkyl nitrites are volatile liquids frequently inhaled to achieve the desired side effects. Amyl nitrite was originally prescribed for the medical management of pain from angina. Once inhaled, it works by dilating blood vessels and increasing the supply of blood and oxygen to the heart while reducing its workload [2]. Inhalation of alkyl nitrites became popular and used by some people to induce an immediate sense of euphoria that can last minutes. Another reason for use is to improve sexual intercourse through the relaxing effects on smooth muscles in the anal sphincter. Alkyl nitrites are easily accessible and legal in the United States (US), often sold as deodorizers or solvent cleaners at adult stores or online. Approximately 3.3% of the US adult population has ever used poppers. A study examining electronic dance festival attendees found that 14.5% had used poppers within the prior year [1].

When poppers are ingested by drinking the volatile liquid, the direct hemoglobin-oxidizing effects of alkyl nitrites induce methemoglobinemia. The literature is limited in case reports regarding alkyl nitrite ingestion. There have been three adults and one pediatric patient reported toxicity from amyl nitrite, all of who presented with severe methemoglobinemia and all successfully treated with methylene blue [3-6]. A literature review of isobutyl nitrite ingestion reveals a similar presentation in adults with severe methemoglobinemia treated with intravenous methylene blue [7-9] and one fatal case of ingestion [7-10]. Although it is not common, early identification of alkyl nitrite-induced methemoglobinemia and quick administration of methylene blue can prevent fatal outcomes. The increasing use of alkyl nitrites (poppers) makes them a drug of abuse and a potential cause of methemoglobinemia when presenting to the ED.

## Case presentation

A 23-year-old female was brought into the ED for altered mental status and acute respiratory failure with hypoxia after being found down at an electronic dance music event. Per event medicine emergency medical services (EMS) who talked to bystanders, before being found down, the patient was seen drinking poppers (alkyl nitrites), as opposed to inhaling them as intended.

The patient initially presented to the event medical tent, where she was evaluated by on-site medical staff, including emergency medicine physicians. The patient was started on intravenous (IV) fluids for hypotension with systolic blood pressures in the 60s millimeters of mercury (mmHg), 15 L non-rebreather for SpO_2_ in the low 80s percentile. She was transported quickly off-site to the ED for concern of methemoglobinemia as the onsite medical tent did not have access to methylene blue.

During EMS transport, the patient had one episode of emesis and had an altered mental status. The patient became less responsive en route. The Glasgow coma scale (GCS) went from 14 to 10 to 4. The patient was hypoxic to 80% on a 15 L nonrebreather. The patient was then ventilated with a bag-valve-mask due to respirations becoming shallow and inadequate. Initial vitals upon arrival to the ED were blood pressure of 96/54 mmHg, slightly hypothermic around 96 degrees Fahrenheit, respiratory rate of 22 breaths per minute, heart rate of 106-156 beats per minute (bpm), and oxygen saturation of 70-80% on a 15 L nonrebreather.

The physical examination was remarkable for cyanosis to digits bilaterally. Pupils were equal to 4 to 3 millimeters reactive bilaterally. Her mental status was GCS 4: GCS eye subscore 1, GCS verbal subscore 1, and GCS motor subscore 2. The cardiopulmonary exam was remarkable for tachycardia and diffuse rales auscultated bilaterally. The abdomen was soft, nontender, and nondistended. There were no obvious signs of trauma and no deformities to bilateral upper or lower extremities.

**Figure 1:**
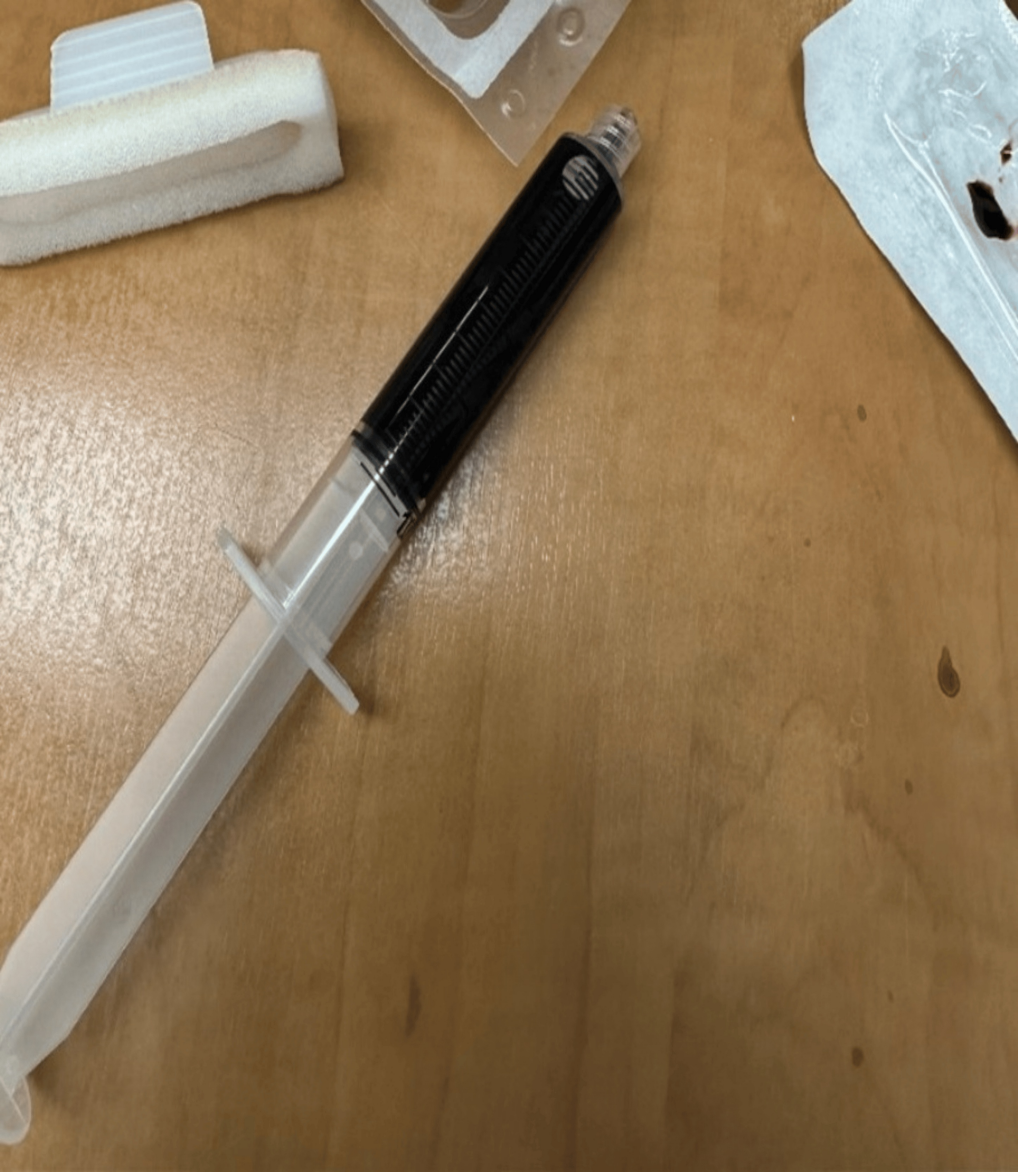
Venous blood sample with chocolate brown color appearance from a patient with poppers-induced methemoglobinemia

Methylene blue was immediately ordered while labs were pending. The patient’s venous blood sample was noted to have a chocolate-brown appearance (Figure 1). Given the low GCS and hypoxemia, the patient was intubated for airway protection while the pharmacy was initiating the methylene blue infusion. Eighty milligrams (mg) of methylene blue was administered as a one milligram per kilogram (mg/kg) dose IV infusion. The patient continued to be hypotensive and required epinephrine pushes and was ultimately started on a Levophed drip for hypotension. A central venous line was placed in the right femoral vein.

Laboratory values of the patient were obtained, as can be seen in Table 1.

**Table 1:** Laboratory values of a patient with methemoglobinemia after orally ingesting poppers

Lab Name	Value	Reference Range & Units
Methemoglobin	Greater than 30.0	0.0-3.0 %
Venous blood gas pH	7.23	7.32-7.43
White blood cells	19.71	4.8-11.8 X10^9^ per liter (bil/L)
Hemoglobin	13.1	12.0-16.5 grams per deciliter (g/dL)
Platelets	278	130-460 bil/L
Prothrombin time	13.1	9.4-12.5 seconds
International normalized ratio	1.1	0.8-1.1
Prothrombin test time	31.3	25.1-36.5 seconds
Sodium	140	134-147 millimoles per liter (mMol/L)
Potassium	3.3	3.5-5.0 mMol/L
Chloride	108	98-109 mMol/L
Carbon dioxide	12	23-32 mMol/L
Anion Gap	20	7-16
Blood urea nitrogen	13	7-20 milligrams per deciliter (mg/dL)
Creatinine	1.2	0.7-1.3 mg/dL
Glucose, Random	109	70-140 mg/dL
Serum osmolality	328	277-297 milliosmoles per kilogram
Lactate	4.7	0.5-2.0 mMol/L
Beta-hydroxybutyrate	0.2	0.0-0.4 mMol/L
High sensitivity troponin	21	Less than or equal to 0.03 nanograms per milliliter
Ethanol	0.165	Less than or equal to 0.010 g/dL
Acetaminophen	Less than 5.0	5.0-27.0 micrograms per milliliter
Salicylate	Less than 0.3	0.0-30.0 mg/dL
Amphetamine	Positive	Negative
Beta-human chorionic gonadotropin qualitative	Negative	Negative

**Figure 2:**
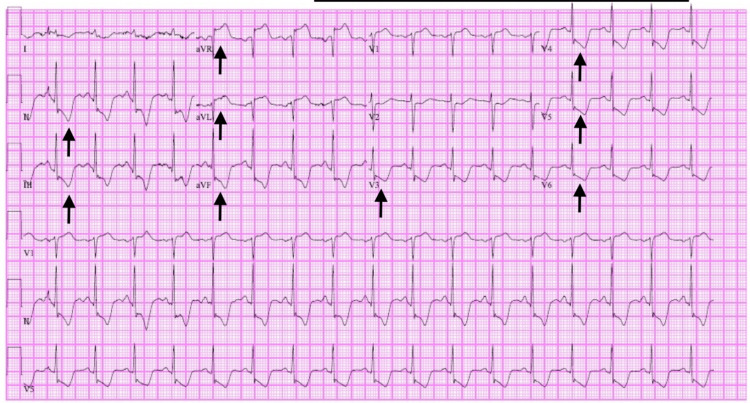
Initial electrocardiogram of a patient with poppers-induced methemoglobinemia showing sinus tachycardia with rate of 104 bpm, and ST-segment elevations in leads AvR and AvL with ST-segment depressions in leads II, III, avF, and V3-V6

Initial electrocardiogram showed sinus tachycardia with a rate of 104 bpm and ST-segment elevations in leads AvR and AvL with ST-segment depressions in leads II, III, avF, and V3-V6 (Figure 2).

**Figure 3:**
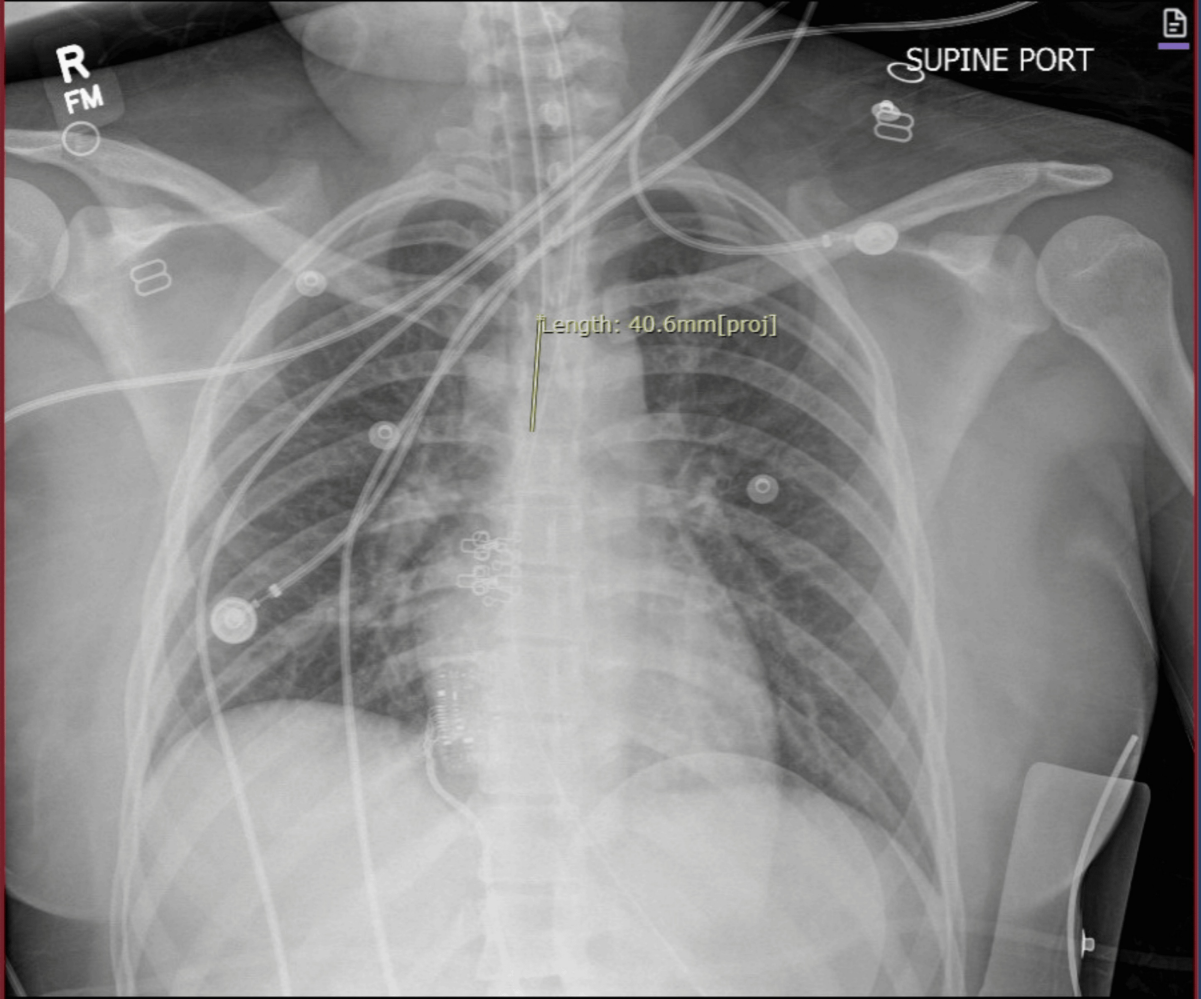
Chest X-ray of a patient with poppers-induced methemoglobinemia after intubation showed no acute abnormality

Cardiology was consulted for consideration of acute coronary syndrome; however, they agreed that ST-segment elevations were likely due to demand ischemia from methemoglobinemia and recommended no role for catheterization lab activation. Chest X-ray after intubation showed no acute abnormality (Figure 3).

**Figure 4:**
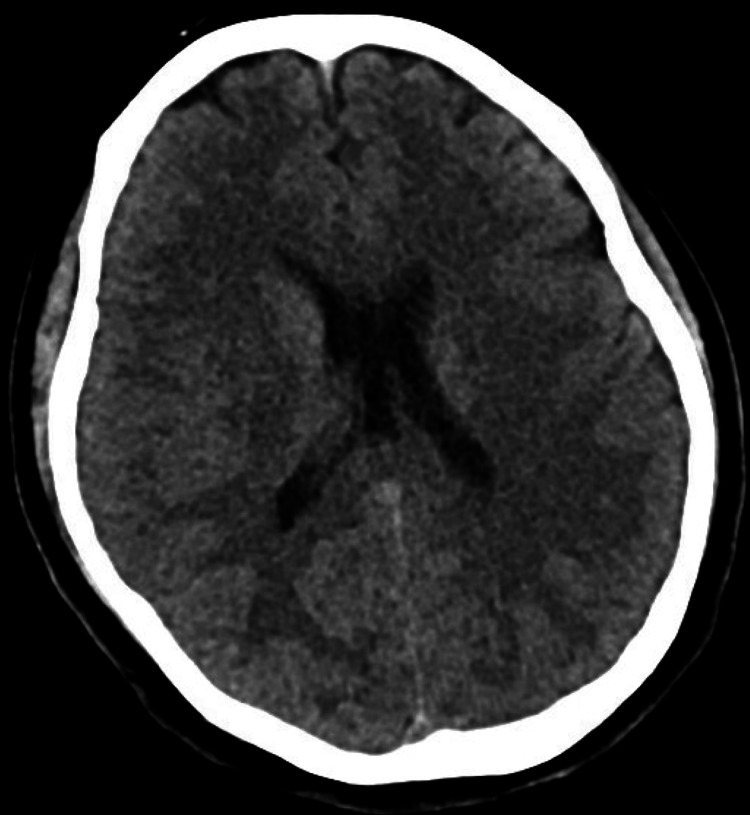
A non-contrast computed tomography (CT) of the head of a patient with poppers-induced methemoglobinemia showed no acute intracranial abnormality

A non-contrast computed tomography (CT) of the head showed no acute intracranial abnormality (Figure 4).

**Figure 5:**
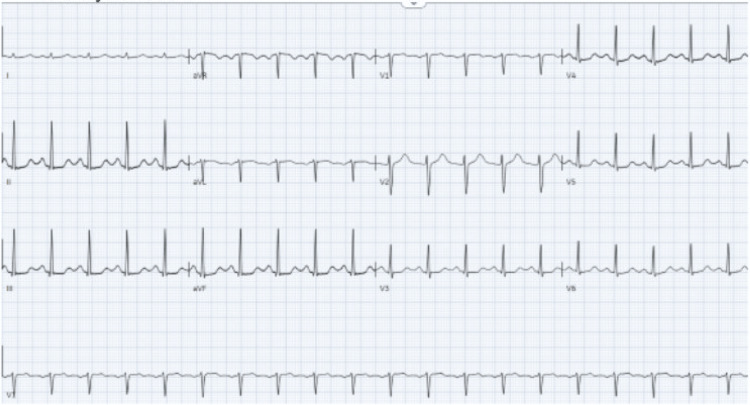
Electrocardiogram of a patient with poppers-induced methemoglobinemia drastically improved after administration of methylene blue, with resolution of ST changes

After the initial 80 mg dose (as a 1 mg/kg dose) of methylene blue was completed, the patient's oxygen saturation improved to the mid-80s. An additional 1 mg/kg dose of methylene blue was ordered. Poison control was contacted and agreed with the treatment of methylene blue. Toxicology was consulted for recommendations given the alkyl nitrite ingestion, and the Medical Intensive Care Unit (MICU) was consulted for admission. Shortly after admission, the patient became alert and wrote her name on paper. ECG drastically improved with the resolution of ST changes (Figure 5).

She was extubated in less than 24 hours, and repeat arterial blood gas on room air showed methemoglobin in the normal range with normal pH, partial pressure of oxygen, partial pressure of carbon dioxide, and bicarbonate. The patient was discharged to home in good health.

## Discussion

It is important to recognize this rare cause of methemoglobinemia in a patient presenting to the ED. Methemoglobinemia is an altered state of hemoglobin in the body. Poppers create methemoglobin through the oxidation of hemoglobin from the normal ferrous (Fe2+) state to the ferric (Fe3+) state. Because the ferric state cannot carry oxygen, this process results in cyanosis. As the amount of methemoglobin rises, the body's natural reducing enzymes become overwhelmed, resulting in symptomatic methemoglobinemia. Classically, methemoglobinemia presents with shortness of breath, cyanosis, headache, tachycardia, and peripheral oxygen saturation of approximately 85%, which does not improve with supplemental oxygen [11].

Clinical effects of methemoglobinemia have been reported as methemoglobinemia 15-30%: cyanosis (tongue, lips, earlobe), fatigue, dizziness, headache; methemoglobinemia 30-50%: weakness, tachycardia, tachypnea, mild dyspnea; methemoglobinemia 50-70%: stupor, coma, convulsions, respiratory depression, cardiac dysrhythmias, acidosis; and methemoglobinemia greater than 70%: potentially fatal [12]. The antidote for methemoglobinemia is methylene blue, given intravenously at a dose of 1-2 mg/kg over five to 30 minutes. Methylene blue mechanism of action is to reduce the oxidized ferric (Fe3+) form of hemoglobin when in a state of methemoglobinemia back to the normal ferrous (Fe2+) state. In turn, this increases the oxygen-binding capacity of hemoglobin and thus increases oxygen delivery to tissues [13].

A characteristic physical finding in methemoglobinemia is chocolate-brown colored blood, which has been seen in other cases [3-5,7,8]. The highest recorded value of methemoglobinemia secondary to the recreational use of alkyl nitrite use is 94% [4].

## Conclusions

It is important to keep poppers overdose on the differential when a patient presents from a music festival or nightlife event with cyanosis, hypoxia, and chocolate-brown blood. Treatment should not be delayed pending lab confirmation in patients who are symptomatic with presumed methemoglobinemia. Early administration of the antidote, methylene blue (as a 1 mg/kg dose), can reverse methemoglobinemia and prevent fatal outcomes from alkyl nitrite ingestion.
